# Retraction: Santacruzamate A Ameliorates AD-Like Pathology by Enhancing ER Stress Tolerance Through Regulating the Functions of KDELR and Mia40-ALR *in vivo* and *in vitro*

**DOI:** 10.3389/fncel.2024.1505362

**Published:** 2024-10-08

**Authors:** Frontiers Editorial Office

The journal retracts the 04 March 2019 article cited above.

Following publication, concerns were raised regarding the integrity of the images in the published figures. The authors failed to provide a satisfactory explanation during the investigation, which was conducted in accordance with Frontiers' policies.

This retraction was approved the Chief Executive Editor of Frontiers. The authors received a communication regarding the retraction and had a chance to respond. This communication has been recorded by the publisher.

